# The impact of different setup methods on the dose distribution in proton therapy for hepatocellular carcinoma

**DOI:** 10.1002/acm2.13178

**Published:** 2021-02-17

**Authors:** Kimihiro Takemasa, Takahiro Kato, Yuki Narita, Masato Kato, Yuhei Yamazaki, Hisao Ouchi, Sho Oyama, Hisashi Yamaguchi, Hitoshi Wada, Masao Murakami

**Affiliations:** ^1^ Department of Radiation Physics and Technology Southern Tohoku Proton Therapy Center Fukushima Japan; ^2^ Preparing Section for New Faculty of Medical Science Fukushima Medical University Fukushima Japan; ^3^ Department of Radiation Oncology Southern Tohoku Proton Therapy Center Fukushima Japan

**Keywords:** diaphragm matching, hepatocellular carcinoma, marker matching, proton therapy

## Abstract

**Purpose:**

To investigate the impact of different setup methods, vertebral body matching (VM), diaphragm matching (DM), and marker matching (MM), on the dose distribution in proton therapy (PT) for hepatocellular carcinoma (HCC).

**Materials and Methods:**

Thirty‐eight HCC lesions were studied retrospectively to assess changes in the dose distribution on two computed tomography (CT) scans. One was for treatment planning (1st‐CT), and the other was for dose confirmation acquired during the course of PT (2nd‐CT). The dose coverage of the clinical target volume (CTV‐D_98_) and normal liver volume that received 30 Gy relative biological effectiveness (RBE) (liver‐V_30_) were evaluated under each condition. Initial treatment planning on the 1st‐CT was defined as reference, and three dose distributions recalculated using VM, DM, and MM on the 2nd‐CT, were compared to it, respectively. In addition, the relationship between the CTV‐D_98_ of each method and the distance between the center of mass (COM) of the CTV and the right diaphragm top was evaluated.

**Results:**

For CTV‐D_98_, significant differences were observed between the reference and VM and DM, respectively (*P* = 0.013, *P* = 0.015). There were also significant differences between MM and VM and DM, respectively (*P* = 0.018, *P* = 0.036). Regarding liver‐V_30_, there was no significant difference in any of the methods, and there were no discernable difference due to the different setup methods. In DM, only two out of 34 cases with a distance from right diaphragm top to COM of CTV of 90 mm or less that CTV‐D_98_ difference was 5% or more and CTV‐D_98_ was worse than VM were confirmed.

**Conclusion:**

Although MM is obviously the most effective method, it is suggested that DM may be particularly effective in cases where the distance from right diaphragm top to COM of CTV of 90 mm or less.

## 1 INTRODUCTION

Hepatocellular carcinoma (HCC) is the fifth most common cancer and second leading cause of cancer‐related deaths in the world.^1^ Since HCCs, which account for most of the primary liver cancers, occur on the background of chronic liver disease, treatment is focused on curative of tumor and preservation of liver function. Although surgery, radiofrequency ablation, and hepatic artery chemoembolization are typical treatments for HCC, radiation therapy has also been reported to have good therapeutic results with advances in treatment techniques such as stereotactic body radiation therapy (SBRT)^2^ or proton therapy (PT).^3^ Due to its excellent dose distribution characteristics, PT can administer high doses to the tumor while minimizing damage to normal liver tissue. Therefore, PT is expected as a new therapeutic method that combines high curability and minimal invasiveness. HCC has been considered as a good indication for PT, and relatively favorable results have been reported mainly in Japan.^3^, ^4^, ^5^, ^6^ By contrast, the target is often adjacent to the intestinal tract, and is susceptible to interfractional motion in addition to intrafractional motion associated from respiratory movement. Therefore, the treatment of HCC is considered to be technically challenging. In recent years, pencil beam scanning (PBS) has been in the spotlight as an irradiation field formation technique for PT,^7^ but careful attention is required for treatment sites with large respiratory movements. Proton therapy with PBS usually shows improved dose distribution and conformity along the proximal edge, however, due to the interplay effect, it is more sensitive to organ motion with respiration than passive scattering PT (PSPT).^8^, ^9^ This suggests that a respiratory‐gated irradiation method using the PSPT still has some useful role. Actually, this method is still widely used in Japan.

In modern radiation therapy techniques such as SBRT or PT for HCC, the vertebral body, diaphragm, and marker (metal marker or lipiodol accumulation) are generally used as indicators of setup depending on the tumor location.^10^, ^11^, ^12^, ^13^ The diaphragm, a typical surrogate for liver motion, is often tracked given its visibility on x‐ray images. The setup performed in our institution performs vertebral body matching (VM) in orthogonal x‐ray imaging, and then performs matching using the diaphragm or marker as an index. In the case of the diaphragm matching (DM), correction is performed only in the superior‐inferior (SI) direction, whereas in the case of the marker matching (MM), three axes of SI, left–right (LR), and anterior–posterior (AP) directions are corrected at our institution. However, especially when using the DM, all parts of the liver does not always move in the same way as the diaphragm because of the large volume of the liver, so the correction may not work effectively depending on the tumor location. In addition, even if it is possible to reduce spatial misalignment by using such an index, proton beams have a finite range, so the water equivalent length of the beam path may change in some cases, the dose coverage for the target and the normal liver dose may be deteriorated. Although there are some reports on the setup using the DM as an index,^11^, ^12^, ^13^ there are no reports on the effect of this method on dose distribution in PT. In addition, the DM is often performed by using on‐board CBCT,^11^, ^12^, ^13^ however, there is no report detailing a method using simple orthogonal x‐ray images that can be implemented at any facility. In this study, we investigated the impact of different setup methods, VM, DM, and MM, on the dose distribution in PT for HCC.

## 2 MATERIALS AND METHODS

### 2.A Patient background

The subjects were 30 patients with 38 HCC lesions treated with PSPT at our institution. Their ages ranged from 24 to 91 yr (mean age, 66 yr). Cases in which a deviation of the right diaphragm of 5 mm or more was observed between the initial planning computed tomography (1st‐CT) and the ongoing verification CT (2nd‐CT) taken under the same conditions for the purpose of confirming reproducibility during the course of PSPT were enrolled in this study. The shift amount was calculated from the difference in the SI direction of the apex of the diaphragm between both CTs. Patient characteristics are summarized in Table 1. This study was approved by the institutional review board of our institution.

**TABLE 1:** Summary of patient characteristics, the displacement of the right diaphragm of the 2nd‐CT with respect to the 1st‐CT and the distance between the COM of the CTV and the right diaphragm top. The diaphragm displacements are positive for movement in the superior direction, and negative for movement in the inferior direction.

Lesion	Gender	Age	Tumor location	CTV (cc)	Diaphragm displacement (mm)	COM‐diaphragm distance (mm)
1	M	71	S8	48	−10	18
2	M	68	S5/7	315	−8	72
3	M	61	S5/6	65	10	85
4	M	24	S2	12	−7	65
5	M	63	S3	49	9	47
6	M	84	S6/7	449	−6	67
7	M	64	S4	23	−8	68
8	M	64	S6	24	−8	90
9	M	66	S6	17	−7	50
10	M	66	S3	5	−7	58
11	M	64	S7	107	−5	73
12	F	62	S6	20	−7	132
13	M	84	S7	22	−8	48
14	M	74	S3/4	71	−6	55
15	M	87	S5/8	177	−8	53
16	M	55	S7	205	−9	67
17	M	59	S1	81	−7	55
18	M	58	S2/3	184	−5	43
19	M	58	S4	48	−5	68
20	F	79	S8	9	15	48
21	F	79	S6	20	−8	83
22	M	60	S7	7	7	25
23	M	60	S4	2	7	70
24	F	53	S4	525	−11	45
25	F	53	S6	29	−11	148
26	M	65	S6	59	7	138
27	M	64	S2	50	13	63
28	F	91	S1	22	9	68
29	F	91	S7	10	9	28
30	F	62	S8	8	−7	62
31	M	59	S7	75	10	53
32	M	55	S5/8	64	−10	70
33	M	71	S8	172	−7	62
34	M	38	S6	51	−6	136
35	M	38	S2	18	−6	62
36	M	65	S5	138	12	88
37	F	78	S8	42	−22	30
38	M	85	S8	9	−9	27
Mean	–	66	–	85	−3	66
SD	–	14	–	118	9	30

Abbreviations: CTV = clinical target volume, M = male, F = female, COM = center of mass, CT = computed tomography, SD = standard deviation.

### 2.B Treatment planning

During simulation, the patients took the supine position with both arms raised and holding handles. A vacuum cushion was used for immobilization of the body. Simulation CT was performed using an Aquilion LB (Canon Medical Systems, Otawara, Japan). For respiratory control, respiratory gated scan during end‐exhalation phase was adopted by applying the respiratory monitoring system, AZ‐733V (Anzai Medical, Tokyo, Japan). The magnetic resonance image (MRI) for PT planning were fused with the 1st‐CT and one physician delineated all contours. Signa HDx (GE Healthcare, IL, USA) was used for MRI. The clinical target volume (CTV) was defined as a three‐dimensional expansion of gross tumor volume (GTV) with 5‐mm margin. The planning target volume (PTV) was defined as additionally a 5‐mm isotropic margin except 7 mm inferiorly aiming in consideration of the movement within the gating window on respiratory movement. The irradiation method was the wobbler method, which is one of the passive scattering methods,^14^ and a plan was created for the beam direction by using the Method described by Moyers et al.^15^ The number of beam angles was determined according to our standard protocol, with two to four angles depending on the tumor location. Although there were no restrictions on the size or location of the target in this study, this is a relative planning study, the prescribed dose was determined as 66 Gy relative biological effectiveness (RBE)/10 fractions at the isocenter for the peripheral type protocol in accordance with the Japanese Society for Radiation Oncology treatment policy of PT (ver 1.0).^16^ The RBE value of 1.1 was used in the present study. Hitachi’s proton‐type Particle Therapy System (Hitachi, Kashiwa, Japan) and XiO‐M (Hitachi, Kashiwa, Japan) were used as the PT machine and the treatment planning system, respectively.

### 2.C Patient setup methods

**FIG. 1:**
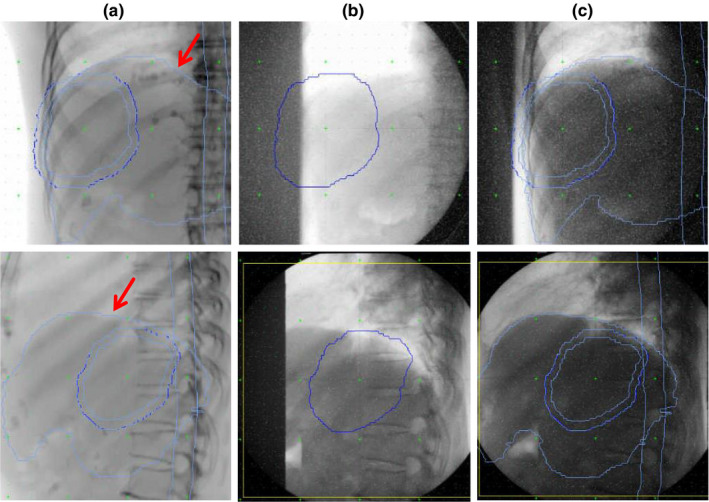
Frontal (upper) and lateral (lower) views during patient positioning: (a) digitally reconstructed radiograph obtained from XiO‐M treatment planning system (RTP), (b) x‐ray imaging during patient setup on vertebral body matching, (c) x‐ray imaging during patient setup on diaphragm matching. Red arrows indicate liver contours delineated on XiO‐M RTP. Other contours in each image show clinical target volume, planning target volume, and spinal canal, respectively.

Patient setup is performed every time immediately prior to beam irradiation using the orthogonal x‐ray imaging system and the six degrees of freedom couch. Our system is not equipped with an in‐room CT or cone‐beam CT (CBCT), therefore, the setup using the vertebral body, diaphragm, and markers as indices is performed for each case based on the orthogonal x‐ray imaging. Figure 1 shows images of the setup in the frontal and lateral views. The contour information delineated using XiO‐M is shown on the digitally reconstructed radiograph (DRR). A specific explanation of each method is given below.

#### 2.C.1 Vertebral body matching (VM)

To remove the setup errors, fine‐tune the six degrees of freedom couch remotely with respect to the reference image using the vertebral body as an index. At this time, the displacement of the liver and respiratory movement are not considered at all.

#### 2.C.2 Diaphragm matching (DM)

The contour information delineated using XiO‐M is shown on the DRR, and this is used as an index for DM. As a procedure, first remove the setup errors by using the orthogonal x‐ray imaging system and the six degrees of freedom couch using the vertebral body as the indices, and then adjust to right diaphragm as the index. At this time, since it is empirically known that the contour of the diaphragm is locally deformed due to the influence of changes in the volume of the large bowel, correction is performed only in the SI direction.

#### 2.C.3 Marker matching (MM)

As a procedure, first remove the setup errors by using the orthogonal x‐ray imaging system and the six degrees of freedom couch using the vertebral body as the indices, and then adjust to marker as the index. Note that correction is performed in the three axes directions.

### 2.D Recalculation procedure on 2nd‐CT

First, in order to copy the CTV to the 2nd‐CT, the 1st‐CT and 2nd‐CT were fused using the anatomical structure around the CTV as the index. Next, the outline of the liver was delineated on the 2nd‐CT as well as the 1st‐CT. We recalculated the dose distributions on the 2nd‐CT after applying the VM, DM, and MM. Each of the actual matching methods described above was simulated using FocalPro (Elekta, Stockholm, Sweden) as follows. The VM was performed so that the vertebral bodies of the 1st‐CT and 2nd‐CT were perfectly matched. The DM was performed to shift the 2nd‐CT in the SI direction so that the right diaphragm was matched after the vertebral bodies of the 1st‐CT and 2nd‐CT were perfectly matched. Thereafter, it was confirmed on the DRR that the right diaphragm was aligned each other. The MM was performed to shift the 2nd‐CT in the three axes directions so that the marker was matched after the vertebral bodies of the 1st‐CT and 2nd‐CT were perfectly matched. In some cases, the marker was not placed near the tumor, but in such cases, the CTV itself was used as an index. Note that the VM has six‐axis correction, but the DM and MM only have translation correction.

### 2.E Analysis

Initial treatment planning on the 1st‐CT was defined as reference plan, and three recalculated dose distributions using VM, DM, and MM on the 2nd‐CT were compared to it. Irradiation conditions were the same as in the reference plan, and D_98_ of the CTV (CTV‐D_98_) and V_30_ of the normal liver (liver‐V_30_) were calculated. Here, CTV‐D_98_ corresponds to the minimum dose required to cover 98% of the CTV. And liver‐V_30_ corresponds to normal liver relative volume that received 30 Gy (RBE). Normal liver was defined as the range of the whole liver minus GTV. Using the CTV‐D_98_ and liver‐V_30_ of the reference plan as a reference, the amount of change on each recalculated dose distribution from that was evaluated. In addition, we analyzed the relationship between the CTV‐D_98_ of each method and the distance between the center of mass (COM) of the CTV and the right diaphragm top. Furthermore, since the subject of this study has a wide range of CTV volume, we also analyzed the relationship between CTV‐D_98_ of each method and CTV volume. A Wilcoxon test was used to determine the statistical significance. *P *< 0.05 were considered statistically significant.

## 3 RESULTS

**FIG. 2:**
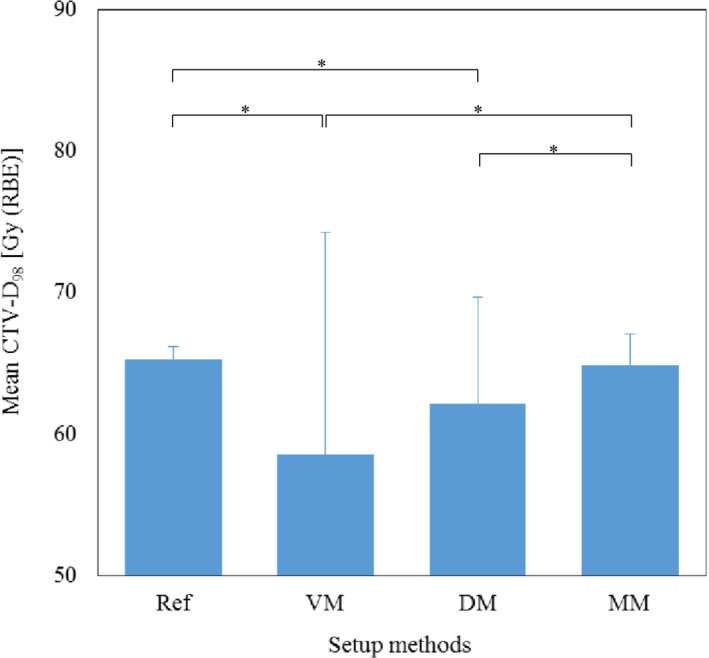
Mean CTV‐D_98_ for each setup method. * *p* < 0.05. Abbreviation: CTV‐D_98_ = the minimum dose required to cover 98% of the clinical target volume, RBE = relative biological effectiveness, Ref = reference, VM = vertebral body matching, DM = diaphragm matching, MM = marker matching.

**FIG. 3:**
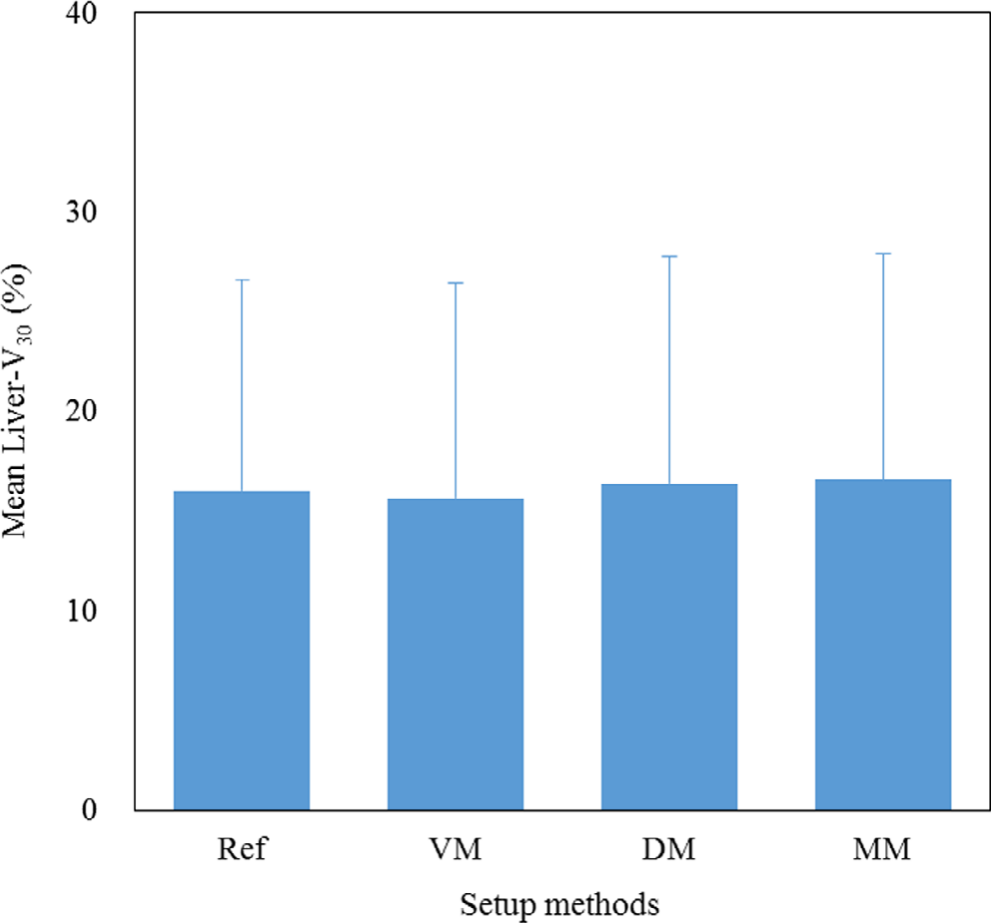
Mean liver‐V_30_ for each setup method. Abbreviation: liver‐V_30_ = normal liver volume which received 30 Gy relative biological effectiveness, RBE = relative biological effectiveness, Ref = reference, VM = vertebral body matching, DM = diaphragm matching, MM = marker matching.

**FIG. 4:**
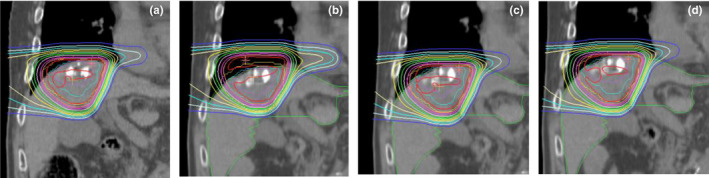
Dose distributions on coronal image for lesion number 1 where there was a displacement of right diaphragm of 10‐mm inferior direction: (a) reference dose distribution on the initial planning computed tomography (1st‐CT), (b) recalculated dose distribution on the ongoing verification CT (2nd‐CT) using vertebral body matching, (c) recalculated dose distribution on 2nd‐CT using diaphragm matching, (d) recalculated dose distribution on 2nd‐CT using marker matching. The dose distribution is displayed on a graduated scale from 10% to 100%.

**FIG. 5:**
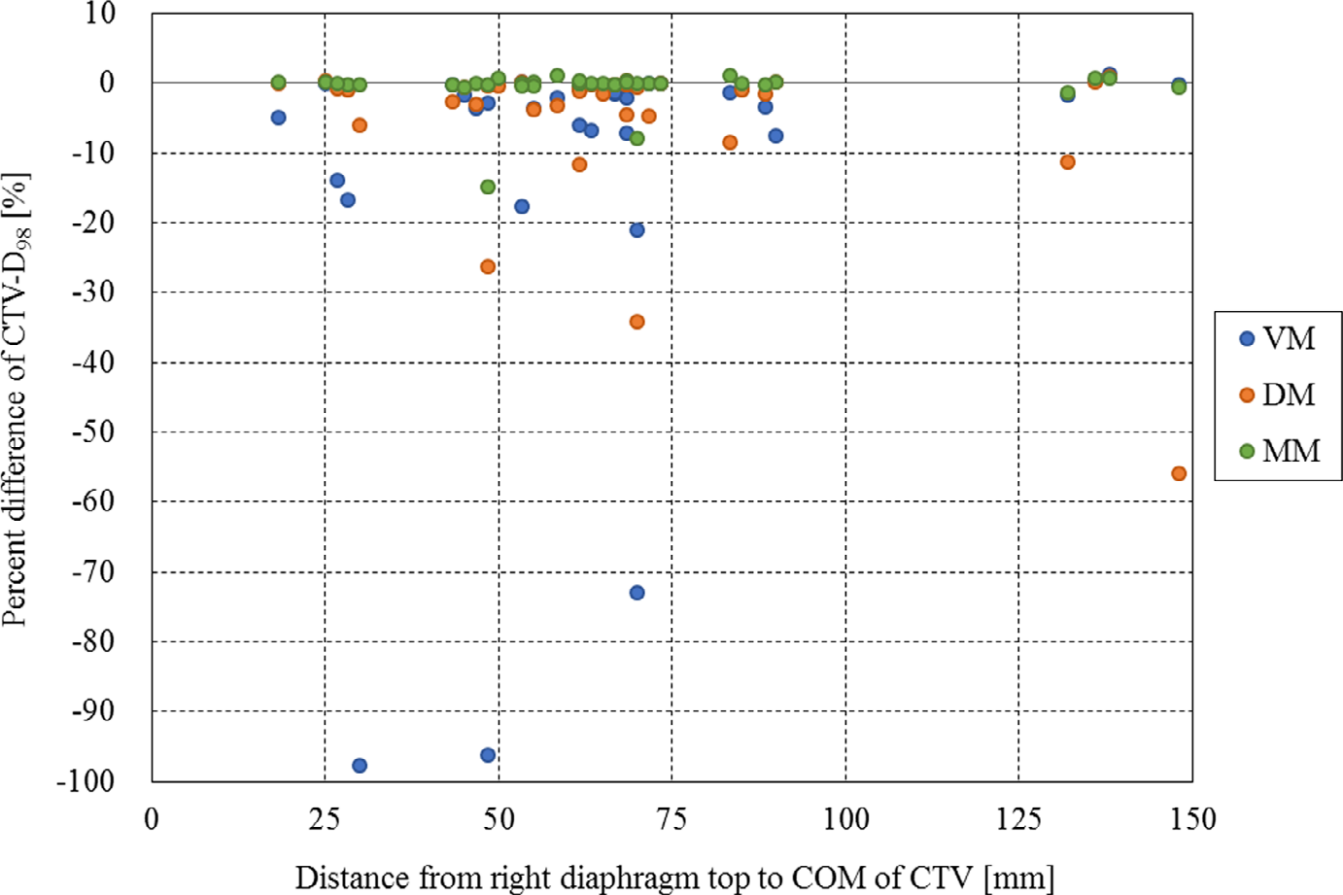
The relationship between percent difference of CTV‐D_98_ and distance from right diaphragm top to COM of CTV. The vertical axis shows the percent difference of CTV‐D_98_ (%), and the horizontal axis shows the distance from right diaphragm top to COM of CTV (mm). The blue, orange, and green points indicate VM, DM, and MM, respectively. Abbreviation: CTV = clinical target volume, CTV‐D_98_ = the minimum dose required to cover 98% of the CTV, COM = center of mass, VM = vertebral body matching, DM = diaphragm matching, MM = marker matching.

**FIG. 6:**
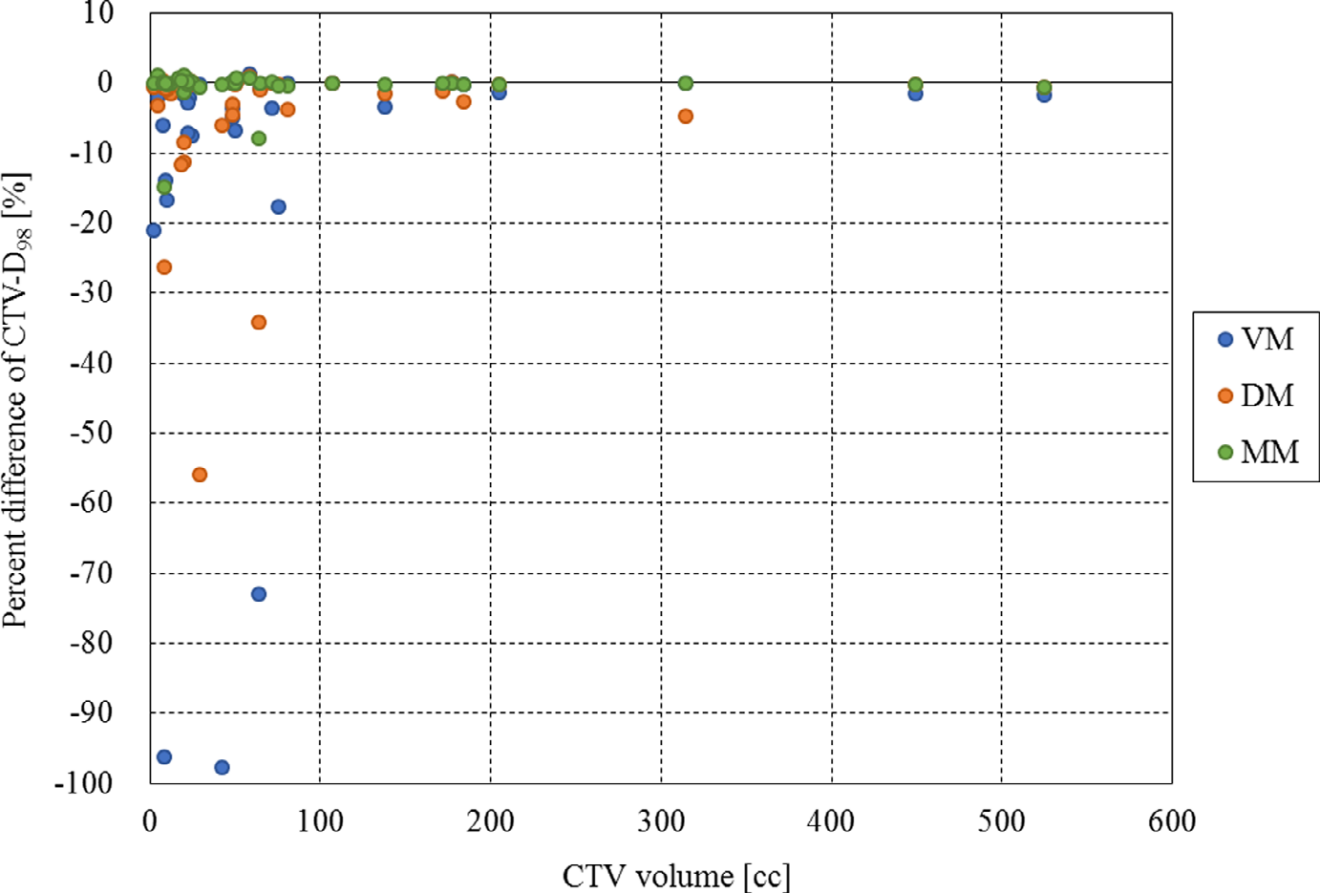
The relationship between percent difference of CTV‐D_98_ and CTV volume. The vertical axis shows the percent difference of CTV‐D_98_ (%), and the horizontal axis shows the CTV volume (cc). The blue, orange, and green points indicate VM, DM, and MM, respectively. Abbreviation: CTV = clinical target volume, CTV‐D_98_ = the minimum dose required to cover 98% of the CTV, VM = vertebral body matching, DM = diaphragm matching, MM = marker matching.

Figure 2 shows the results of mean CTV‐D_98_ for each method. Significant differences were observed between the reference and VM and DM, respectively (*P* = 0.013, *P* = 0.015). There were also significant differences between MM and VM and DM, respectively (*P* = 0.018, *P* = 0.036). Figure 3 shows the results of mean liver‐V_30_ for each method. No significant difference was found between all methods in liver‐V_30_. Figure 4 shows an example of the dose distribution in lesion number 1 obtained by matching each index. In cases where there was a displacement of the right diaphragm of 10‐mm inferior direction, it can be seen visually that the dose distribution has changed depending on each setup method. Almost no change was observed in DM and MM, but in VM, it can be seen that the upper side of the irradiation field with the lateral beam did not deposit its energy in the liver and the exposure to the heart was increased. CTV‐D_98_ difference against the reference in this case was less than 0.3% in DM and MM, while it decreased by approximately 5% in VM. Table 1 summarizes the results of the displacement of the right diaphragm of the 2nd‐CT with respect to the 1st‐CT and the distance between the COM of the CTV and the right diaphragm top. Figure 5 shows the relationship between percent difference of CTV‐D_98_ and the distance from the right diaphragm top to the COM of the CTV. It can be seen that 29 out of 38 cases (76%) exist between the distances from the right diaphragm top to the COM of the CTV of 25 and 75 mm. Of these, CTV‐D_98_ difference of <5% was observed for 19 cases (66%), 25 cases (86%), and 27 cases (93%) for VM, DM, and MM, respectively. CTV‐D_98_ difference of more than 5% in MM was observed in two cases. It was confirmed that these were cases of increased ascites and cases of extremely irregular target shape, respectively. In addition, when viewed as a whole, CTV‐D_98_ difference of less than 5% was observed for 26 cases (68%), 31 cases (82%), and 36 cases (95%) for VM, DM, and MM, respectively. The shorter the distance from the right diaphragm top to the COM of the CTV, the more likely the CTV‐D_98_ underdose in VM. It was found that CTV‐D_98_ in DM was worse than VM in four of seven cases in which CTV‐D_98_ difference in DM was not less than 5%. It was confirmed that CTV was located in S6 in three of these four cases. In addition, in DM, only two out of 34 cases with a distance from right diaphragm top to COM of CTV of 90 mm or less the CTV‐D_98_ difference was 5% or more and CTV‐D_98_ was worse than VM were confirmed. Figure 6 shows the relationship between percent difference of CTV‐D_98_ and the CTV volume. From this result, it can be seen that the deterioration of CTV‐D_98_ by 5% or more is limited to the case where the CTV volume is 75 cc or less regardless matching method.

## 4 DISCUSSION

Although there are many reports on the setup method in SBRT for liver cancer,^11^, ^12^, ^13^ there are few reports in PT. It is often observed that the diaphragm location changes from the time of the 1st‐CT during the course of PSPT for HCC. We examined the dosimetric effect of different setup methods on dose distribution using 2nd‐CT data that showed a right diaphragm position difference of 5 mm or more in CT images taken during the course of PSPT for the purpose of confirming position reproducibility. We investigated the effects of the CTV dose coverage and normal liver exposure for the VM, DM, and MM, which are setup methods that can be performed independently of PT machine specifications. In this study, the PTV margin was set to be relatively tight, a 5‐mm isotropic margin except 7 mm inferiorly. In the case of VM and DM, the number of cases that the dose coverage of CTV‐D_98_ could not meet the 95% dose, which is our criterion, was in 12 (32%) and 7 (18%), respectively, but only two cases (5%) in MM. This suggests that MM is obviously the best matching method, however, if an appropriate setup method is selected according to the case, a certain degree of accuracy can be also ensured even in DM. It is widely known that liver motion is highly affected by respiration, with the largest induced motion usually observed in the SI direction.^17^ The diaphragm shape visible on the two‐dimensional (2D) x‐ray images often appears deformed from the time of the 1st‐CT, but it may locally occur due to the change in the intestinal volume or other reason. In such cases, there is a possibility that overcorrection may occur if DM is performed in all directions. We considered that this pitfall should be taken into consideration when selecting DM in all directions. Therefore, in DM according to our institutional standard protocol, only the correction in the SI direction is performed with the understanding that residual error remains. It is ideal to place a plurality of markers near the target and perform MM using these markers as an index. However, in practice, it is actually difficult to perform this in all cases. Therefore, it is desirable to consider in advance how to deal with cases where there is no clear index such as a metal marker or lipiodol accumulation. In particular, when the CTV size is relatively small and localized in the S8, if VM is adopted when the displacement of the right diaphragm is 10 mm or more, the coverage may be extremely deteriorated. Since the dose gradient of PT is steep, cold spots are likely to occur in a part of the CTV if spatial misalignment beyond the safety margin occurs. And the smaller the CTV volume, the greater the impact on dose coverage. It is considered that this is the reason why there were two cases where the displacement of CTV‐D_98_ difference was close to −100% when using VM. In some cases, the significance of treatment itself may be lost, so sufficient caution is required. Therefore, DM or MM adoption or PTV margin expansion should be considered in such case.

On the other hand, although there are few cases, it is suggested that the accuracy may not be ensured even with MM. In addition, it was suggested that it is necessary to recognize that liver‐V_30_ may deviate from the evaluation value of the initial treatment plan by 10% or more in all matching methods. Therefore, it is desirable to check the condition in the abdomen by taking CT images regularly for more safety. Especially in the case of close proximity to the intestinal tract, changes often cannot be captured only by checking the diaphragm or markers on 2D x‐ray images, and it is considered that confirmation by CT is more important in such cases. Hawkins et al. evaluated residual errors using CBCT after setup using two orthogonal MV imaging, and reported that 33% of deviations of 5 mm or more were observed.^18^ In addition, although it was small, displacement of 10 mm or more and deformation of 5 mm or more were also observed. Since high‐dose prescriptions are usually adopted in PT, it is more important to perform the therapy on the assumption that such problems may occur.

It was found that CTV‐D_98_ in DM was worse than VM in four of seven cases in which CTV‐D_98_ difference in DM was not <5%. It was confirmed that CTV was located in S6 in three of these four cases. Therefore, care must be taken when applying the DM to the CTV existing in S6. Kawahara et al. reported that the movements of the diaphragm and the target were in good agreement with each other in a study on breath‐holding irradiation,^12^ but the results of this study suggest that this is not always the case. By contrast, Yang et al. evaluated the relationship between liver tumor motion and diaphragm motion in 14 liver cancer cases,^19^ and concluded that tumor and diaphragm motions had high concordance when the distance between the tumor and tracked diaphragm area was small. An early study using animal models with implanted markers at different locations within the liver indicated that the motion magnitude of the liver varied with the distance between the diaphragm and the measurement point.^20^ These results are considered to be consistent with our result in this study. In addition, in DM, only 2 out of 34 cases with a distance from right diaphragm top to COM of CTV of 90 mm or less that CTV‐D_98_ difference was 5% or more and CTV‐D_98_ was worse than VM were confirmed. Therefore, it was suggested that DM can be an effective method if the distance from the right diaphragm top to COM of CTV is 90 mm or less in the case where MM cannot be used. In this study, all DM used the right diaphragm as an index, but the left diaphragm might be better as an index on the left lobe. This point is considered as a subject for future study.

In recent years, PBS has become widespread. Although there is a merit that the conformity on the proximal side can be improved compared to PSPT, there are also problems such as the risk that an interplay effect may occur for moving targets. Despite a retrospective analysis, Yoo et al. analyzed the outcomes of both methods and reported that they were comparable.^21^ The problem of positioning in HCC is a common issue for both PSPT and PBS, and the results of this study are expected to be a useful data for PBS, which will become mainstream in the future.

In many cases, since HCC does not have a target contrast on an x‐ray image unlike lung cancer, position matching is an issue. In addition, it is not always easy to safely prescribe a high dose because the respiratory movement is large and the intestinal tract is often present in close proximity. Because the left liver is affected by changes in the volume of the stomach and the right liver is affected by changes in the volume of the large bowel, we default to 4 h of fasting before treatment. Nevertheless, it is difficult to completely control the contents of the intestinal tract, and it is often experienced that the gas in the stomach and large bowel is so substantial that the treatment must be interrupted. In addition, when the intestinal tract is located close to the target caudal side, the gate level and caudal internal margin in respiratory‐gated irradiation often have to be set tightly. Various changes can occur during the course of PT for HCC, such as changes in the position of the intestinal tract, changes in the volume of the liver, and increases in ascites.^22^ These changes can cause serious adverse events, especially when prescribing high doses, so care must be taken. Specifically, it would be desirable to evaluate the adequacy of continuing the treatment with each setup method by taking CT images at appropriate timing during the course of PT. In order to continuously carry out this in daily practice, it would be ideal to perform three‐dimensional setup with an in‐room CT or CBCT. Since there is a problem with image quality in CBCT to check the condition of the intestinal tract and ascites, it would be most ideal to have an in‐room CT capable of high‐speed imaging. On the other hand, it is not uncommon for baseline shift or drift to occur during irradiation,^23^ and considering that it is necessary to confirm and correct them as necessary. Therefore, the use of MM may be reasonable when considering efficient treatment. A high‐precision tumor setup can be established shortly before dose delivery, which minimizes the impact of tumor drifts. To summarize the above contents, it can be said that the most reliable treatment tactic is to mainly setup with MM and check the details with in‐room CT regularly or as needed. The ultimate form seems to be an MR guided,^24^ but it will take more time to put it to practical use in PT. Actually, required setup accuracy depends on the prescribed dose, the distance between the CTV and the intestinal tract, and the setting of the safety margin for the CTV, so the optimal matching method is not uniquely determined. However, SBRT and PT, which prescribe doses that exceed the tolerance dose of the intestinal tract, require careful consideration at each institution, and this study can help in the case of making a decision in actual clinical practice.

## 5 CONCLUSION

The impact of different setup methods on the dose distribution in PT for HCC was evaluated. Although MM is obviously the most effective method, it is suggested that DM may be particularly effective in cases where the distance from right diaphragm top to COM of CTV of 90 mm or less. However, if the CTV volume is small, careful handling will be required. On the other hand, liver PT is susceptible to changes in the intestinal tract, changes in the volume of the liver, and increased ascites in addition to respiratory movements, and none of the setup methods can completely solve these problems. Moreover, the efficacy of each setup method is highly variable between patients. Therefore, it would be desirable to conduct CT scans during the course of PT and confirm the validity of continuing the treatment using the adopted setup method. Although it is difficult to find a single clear index that can be used as a reference when deciding on an appropriate setup method, the results of this study can help in the case of making a decision in actual clinical practice.

## AUTHOR CONTRIBUTION STATEMENT

Kimihiro Takemasa: Conceptualization, Methodology. Writing–Original Draft. Takahiro Kato: Conceptualization, Investigation, Writing–Original Draft. Yuki Narita: Resources. Masato Kato: Formal analysis. Yuhei Yamazaki: Formal analysis. Hisao Ouchi: Validation. Sho Oyama: Validation. Hisashi Yamaguchi: Methodology, Validation. Hitoshi Wada: Investigation, Writing–Review & Editing. Masao Murakami: Supervision, Writing–Review & Editing.

## CONFLICT OF INTEREST

The authors declare no conflict of interests.
